# Novel bivalent transition metal complexes based on a 2-amino-3-hydroxypyridine Schiff base ligand: synthesis elucidation, antimicrobial evaluation, antioxidant and molecular docking studies

**DOI:** 10.1186/s13065-025-01561-8

**Published:** 2025-07-01

**Authors:** A. Z. El-Sonbati, A. A. El-Bindary, N. M. Mansour, M. M. El-Zahed

**Affiliations:** 1 Chemistry Department, Faculty of Science, Damietta University, Damietta, Egypt; 2 Botany and Microbiology Department, Faculty of Science, Damietta University, P.O. Box 34517, New Damietta City, Egypt

**Keywords:** Schiff basecomplexes, Antimicrobial, Molecular docking, Minimum inhibition concentration, Minimum microbicidal concentration

## Abstract

Cu(II), Co(II), Ni(II), Mn(II), and UO_2_(II) complexes have been prepared and studied using a Schiff base generated from 2,4-dihydroxybenzaldehyde and 2-amino-3-hydroxypyridine. Various spectroscopic methods have inferred the complexes' shape and bonding type. The Schiff base and its metal complexes were examined for antibacterial activity against bacteria including *Pseudomonas aeruginosa*, *Bacillus cereus*, *Escherichia coli*, and *Staphylococcus aureus*, as well as fungi such *as Fusarium oxysporum*, *Aspergillus niger*, and *Candida albicans*. The in vitro antimicrobial assay was conducted using the agar well diffusion method, minimum inhibition concentration (MIC), and minimum microbicidal concentration (MMC) tests. All prepared compounds demonstrated effective inhibition potential against the selected harmful fungi compared to their antibacterial activity. The antioxidant assay utilizing the DPPH method indicated that Mn(II), Cu(II), Co(II), and Ni(II) complexes were the most active compounds, showing DPPH radical scavenging activities of 76.2, 68.4, 65.3, and 60.1% inhibition, respectively. This study also evaluated the molecular docking performance and interaction mechanisms of the ligand and its metal complexes against three fungal targets: *C. albicans* (PDB ID 5V5Z), *A. niger* (PDB ID 3PL3), and *F. oxysporum* (PDB ID 1FN8). Docking scores (S), interaction energies, and refined RMSD values were calculated. Results revealed that complex (3) exhibited the strongest binding affinity against *C. albicans* (S = −9.28784), while complex (5) showed notable interactions with *F. oxysporum*. Key interactions included hydrogen bonds, π–H, and π-cation interactions, with energies reaching as low as −4.4 kcal/mol. These findings highlight the potential of metal-based complexes as antifungal agents. The results demonstrated that the Schiff base and its metal complexes possess promising antimicrobial activity, which may be beneficial for pharmaceutical and industrial applications.

## Introduction

The defensive mechanisms that microbes have evolved to withstand the effects of antibiotics or chemicals are known as antimicrobial resistance (AMR) [1]. Fungi and bacteria are among the many microorganisms that can develop such resistance to antimicrobial treatments that they become ineffective. Recently, Gram-positive bacteria such as *Bacillus cereus* and *Staphylococcus aureus*, along with Gram-negative bacteria like *Escherichia coli* and *Pseudomonas aeruginosa*, as well as fungi including *Aspergillus* sp., *Fusarium*, and *Candida* sp., have demonstrated resistance behaviors against various chemical classes [2–4]. Different microbial resistance mechanisms, including efflux systems, target alteration, resistance genes, endospore formation, biofilm formation, and others, are employed and developed by microorganisms to evade the antimicrobial action of several current ligands and metal complexes [5, 6]. Therefore, great attention and research are being devoted to designing and fabricating new antimicrobial agents with distinct properties [7–9].

As promising antimicrobial agents, ligands containing both azo and azomethine groups within the same structure are known as azo Schiff bases. In coordination chemistry, Schiff bases serve as important ligands due to their remarkable donor qualities, ease of synthesis, and high solubility in common solvents [10, 11]. Given the excellent stability of coordination compounds, Schiff bases have found extensive applications as ligands. In Schiff bases, the π-system often imposes geometrical constraints and influences the electronic structure. They have been reported to exhibit a variety of activities, including the detection of malignancies and the activity against bacteria and fungi. The biological activity of these azo-containing Schiff bases is noteworthy, encompassing antifungal, antibacterial, food, leather, textile, pharmaceutical, and cosmetic applications [12–14]. Since azo-base compounds can form complexes with transition metal ions that bind to DNA bases by coordinating bonds with the nitrogen atoms in the DNA bases to create a chelate ring, their potential as antibacterial agents has been established in the medical field [15, 16]. However, Alayyafi et al. [15] reported the microbial resistance of *Fusarium oxysporum*, *Aspergillus niger*, and *Candida albicans* to various metal(II) acetate complexes prepared with a heterocyclic azo dye ligand. Thus, new azo Schiff bases are necessary to combat this microbial resistance [17, 18].

Transition metal complexes with hydroxypyridine ligands, which form when 2,4-dihydroxybenzaldehyde condenses with an amine, have been the focus of extensive research recently. Metal complexes of hydroxypyridine and related Schiff bases are popular in research due to their importance as biomimetic catalysts in the oxygenation process [19]. The unique characteristics of these compounds stem from their bonding mechanisms and chelating ability with the core metal atom. For coordination to occur, the imine group (–N=CH–) acts as a potential donor. These versatile ligands exhibit a range of biological properties and chelating activities.

The Schiff base ligand (HL) was synthesized in this study by condensing 2-amino-3-hydroxypyridine and 2,4-dihydroxybenzaldehyde. It was then treated with Cu(II), Co(II), Ni(II), Mn(II), and UO_2_(II) acetates to produce five metal complexes. A variety of analytical methods were used to characterize these complexes. Several heterocyclic Schiff base ligands and their transition metal complexes have been actively studied for their interesting coordination chemistry and biological applications, including antimicrobial activities, anticancer potential, and other medicinal uses [20–25]. These include complexes with Cu(II), Co(II), Ni(II), Zn(II), Mn(II), UO_2_(II), and Ru(II) and Schiff base ligands derived from 2-amino-3-hydroxypyridine and 2,4-dihydroxybenzaldehyde [26]. Aromatic aldehydes often generate ligands through condensation reactions more quickly than ketones. Compared to aromatic aldehydes, aliphatic aldehyde and ketone Schiff base ligands are less stable due to the presence of effective conjugation in the ring system. The presence of a donating substituent at the ortho position facilitates coordination, and the nitrogen atom of the azomethine group’s sp2 hybridized orbital contains a lone pair of electrons that significantly contribute to coordination and provide good chelating capabilities. In an ethanolic solvent, aliphatic or aromatic aldehydes condense with primary aliphatic or aromatic amines to form Schiff base ligands. The reaction is accelerated by adding a catalytic amount of acid [27].

The antimicrobial and antioxidant properties of the Schiff base and its metal complexes were among the biological activities examined against various microbial strains using the agar well diffusion method, minimum inhibitory concentration (MIC), and minimum microbicidal concentration (MMC). The produced Schiff base and its metal complexes were also subjected to molecular docking studies to evaluate their antimicrobial qualities, which may help in understanding their antimicrobial mechanisms.

## Materials and instruments

### Materials

All chemicals were of analytical reagent grade and used without further purification. 2,4-dihydroxybenzaldehyde and 2-amin-3-hydroxypyridine were purchased from Aldrich Chemical Company. The chemicals obtained from Sigma included the metals Cu(CH_3_COO)_2_·H_2_O, Co(CH_3_COO)_2_·2H_2_O, Ni(CH_3_COO)_2_·2H_2_O, Mn(CH_3_COO)_2_·4H_2_O, and UO_2_(CH_3_COO)_2_·2H_2_O. As previously stated, all measures, techniques, and equipment were utilized [28–33].

### Methods

#### Synthesis of Schiff base and its complexes

**Fig. 1:**
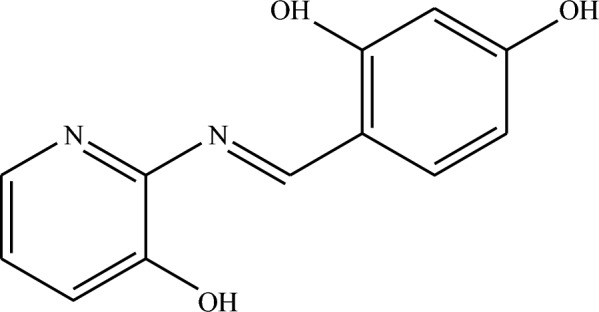
The structure of the Schiff base ligand (HL)

Figure 1 illustrates the structure of the Schiff base ligand (HL). An air-stable colored solid with a good yield (84–78%) was produced by the complexes formed throughout refluxing for 3–5 h, through magnetic stirring of an ethanol solution containing Schiff base and metal acetates in a 2:1 molar ratio. After filtration, the precipitate was collected and thoroughly washed with ethanol. Table 1 presents the analytical FT-IR, electronic spectroscopy, magnetic moment, and molar conductance of the metal complexes.

**Table 1:** Analytical FT-IR, electronic spectroscopy, magnetic moment, and molar conductance of complexes

Compound	FT-IR (cm^−1^)	λ_max_ (cm^−1^)	μ_eff_ (BM)	Molar conductance (Ohm^−1^ cm^−2^ mol^−1^)	Elemental analysis Calcd. (Found) (%)		
					C	H	N
[Cu(L)_2_(OH_2_)_2_]2H_2_O (1)	υ(C=N)1532, υ(OH)3241, υ(C–O)1232	17,240	1.72	18	51.66 (51.54)	3.55 (3.45)	11.44 (11.44)
[Co(L)_2_(OH_2_)_2_]3/2H_2_O (2)	υ(C=N)1551, υ(OH)3330, υ(C–O)1223	15,408 17,677 27,027	4.36	23	49.66 (49.54)	3.79 (3.57)	9.66 (9.36)
[Ni(L)_2_(OH_2_)_2_]0.5H_2_O (3)	υ(C=N)1593, υ(OH) 3330, υ(C–O)1220	22,195 20,156 10,345	2.99	21	51.27 (51.13)	3.92 (3.82)	9.97 (9.67)
[Mn(L)_2_(OH_2_)_2_] (4)	υ(C=N)1550, υ(OH) 3322, υ(C–O) 1228	15,560 21,478 27,876	5.44	12	52.47 (52.36)	4.01 (3.81)	10.20 (9.72)
[UO_2_(L)_2_] (5)	υ(C=N)1544, υ(OH)3325, υ(C–O) 1218	22,970	Dia.	10	39.56 (39.49)	2.47 (2.37)	7.69 (7.46)

#### Antimicrobial potential using the agar well diffusion method

*F. oxysporum*, *A. niger*, and *C. albicans* were utilized to screen for antifungal activity, while various bacterial strains, including *B. cereus* and *S. aureus* (Gram-positive bacteria), as well as *P. aeruginosa* and *E. coli* (Gram-negative bacteria), were employed to assess antibacterial activity [34]. The Clinical and Laboratory Standards methodology was adhered to for conducting the antimicrobial testing [35]. Three concentrations of the Schiff base and its complexes (50, 100, and 150 µg/ml) were prepared in dimethylformamide (DMF) and tested against the selected microbial strains. Conventional antibacterial and antifungal medications, such as penicillin G and miconazole, were also examined. DMF was included to measure the zone of inhibition (ZOI) of the Schiff base and its metal complexes (ZOI = ZOISchiff base or Complex – ZOIDMF). Each experiment was performed three times.

#### Minimum inhibition concentration

Two sets of conical flasks were utilized to prepare the nutrient broth medium (NBM), which was inoculated separately with a 0.5 McFarland standard (1–2 × 10^8^ CFU/ml) from each microbial strain, using varying doses (10–150 µg/ml) of Schiff base and its metal complexes. Similarly, DOX broth medium flasks were employed to evaluate fungal strains [36, 37]. For bacteria or fungi, the inoculation flasks were incubated for 24 h or 5 days at 37 °C or 30 °C, respectively. To determine the MIC values, growth was monitored spectrophotometrically at 600 nm.

#### Minimum microbicidal concentration

Using the pour plate technique, the MIC flasks were inoculatedwith the Schiff base and its metal complexes (including 4 concentrations ≥ MIC) onto nutrient agar medium (NAM) and DOX agar plates. The bacteria and fungi were then incubated for 24 h and 5 days at 37 and 30 °C, respectively [38, 39]. The total number of microbial colonies in CFU/ml (colony-forming unit/ml) were examined and calculated. MMC values were recorded at the complete inhibition of microbial growth (clear plates).

#### Antioxidant characteristics

The 2,2-diphenyl-1-picrylhydrazyl (DPPH) free radical scavenging activity method was used to examine the radical scavenging capabilities of the synthesized Schiff base and its complexes [40]. The highest absorbance of DPPH radical solutions occurs at 517 nm. To prepare the solution, a 1 mM DPPH radical solution was prepared, and ethanol was used to reduce the control sample's absorbance to 1.5 ± 0.5. Standard antioxidant ascorbic acid (vitamin C) was employed for outcome comparison. The DPPH radical solution was administered separately to the samples and standards at varying doses (5–100 µg/ml). After a 30-min incubation period at 25 °C, the absorbance values of the samples were measured. A sample's ability to scavenge DPPH free radicals is indicated by its decreased absorbance at 517 nm. The inhibition of DPPH in percent (I %) was calculated according to % = (A_control_ − A_compound/Acontrol_) × 100, where A_control_ is the absorbance when the compound is absent, and A_compound_ is the absorbance when the compound is present.

#### Molecular docking measurements

MOE (Molecular Operating Environment, Montreal, Chemical Computing Group Inc., QC, Canada) software version 2019 was used for molecular docking [41]. The protein structures of *C. albicans* (PDB ID 5V5Z), *A. niger* (PDB ID 3PL3), and *F. oxysporum* (PDB ID 1FN8) were obtained from the PDB, protonated, and energy-minimized using the AMBER10: EHT force field [42]. Ligand N and its metal complexes were prepared by optimizing their 3D geometries and assigning partial charges using the MMFF94x force field for docking into the active sites of the fungal crystal structures [41]. The parameters included the London dG scoring function for initial placement, refinement through force field energy minimization, and final scoring with the GBVI/WSA dG method [43]. The RMSD refinement threshold was set to 2.0 Å. Interaction analyses (hydrogen bonds, π-effects) were conducted using MOE’s pose analyzer, with energy terms recorded to assess conformational stability and binding efficacy: Energy conformer score (E_conf), energy placement phase score (E_place), and energy refinement score (E_refine) [44, 45].

#### Data analysis

The mean ± standard deviation (SD) is the standard error of the mean. Experimental data were presented as mean ± SD and analyzed using SPSS 19.0. ANOVA was conducted for MIC/MMC and antioxidant tests. The *p* ≤ 0.05 significance threshold was applied.

## Results and discussion

### Synthesis and characterization

At room temperature, all complexes are stable solids that dissolve in DMSO and DMF. Both elemental analysis and FT-IR spectral measurements confirmed the purity of the Schiff base. In contrast to the observed values of C, 62.97; H, 4.22; N, 11.75 percent, the microanalytical values for HL, which were determined using the empirical formula C_10_H_8_N_2_O_2_, are C, 62.61; H, 4.35; N, 12.17 percent. The complexes in DMF were dissolved in 10-3 M solutions to measure the molar conductance at room temperature. The non-electrolytic character of all complexes is supported by the values [46]. The experimental section presents the analytical data of the complexes.

Despite the complexity of the complexes' infrared spectra, significant bands have been identified based on their relationships to other comparable complexes [16, 47–49]. The spectra of the complexes also contained the frequency attributed to O–H vibrations, as shown in the HL spectrum (3315 cm^−1^). This indicated the presence of a single –OH group. This discovery verified that a metal ion replaced the proton of one –OH group during complexation [47, 50], while another -OH group remained unaltered. The C=N stretching vibrations responsible for the sharp band observed at 1564 cm^−1^ in the HL spectrum were altered in the spectra of the complexes. Additionally, this observation implied that C=N was involved in the complexation [33, 47].

### Agar well diffusion investigation

The findings of the agar well diffusion method are presented in Tables 2 and 3. DMF exhibited no inhibition zones against any of the tested microorganisms. Compared to other chemicals, the Ni(II) complex displayed the strongest antibacterial activity against both Gram-positive and Gram-negative bacteria. These results aligned with those of Salehi et al. [51]. With a ZOI of 12, 15, 8, and 9 mm, respectively, *E. coli* was the most susceptible bacteria to 150 µg/ml of Cu(II), Ni(II), Mn(II), and UO_2_(II) complex treatments. Additionally, Cu(II) and Ni(II) complexes exhibited a 10 mm inhibitory zone at 150 µg/ml, demonstrating a strong antibacterial effect against the drug-resistant bacterium *P. aeruginosa*. Furthermore, the Ni(II) complex surpassed the common antimicrobial penicillin G in terms of antibacterial activity against both *E. coli* and *P. aeruginosa*. While most of the complexes under investigation lacked the potency of the Ni(II) complex, several of them showed efficacy against *E. coli*. *S. aureus* exhibited complete resistance against the Mn(II) complex, while P. aeruginosa was resistant to the Co(II) complex. The compound with the strongest antibacterial activity was the Ni(II) complex, followed by the Cu(II) and UO_2_(II) complexes.

**Table 2:** Antibacterial activity of Schiff base and their metal mixed Schiff base complexes

Compound	Concentration (µg/ml)	Gram-positive bacteria		Gram-negative bacteria	
		*Bacillus cereus*	*Staphylococcus aureus*	*Escherichia coli*	*Pseudomonas aeruginosa*
Ligand	50	0	0	0	0
	100	0	6 ± 0.06	0	0
	150	7 ± 0.03	8 ± 0.06	7 ± 0.14	6 ± 0.14
Cu(II)	50	0	0	6 ± 0.03	6 ± 0.06
	100	0	0	9 ± 0.03	8 ± 0.06
	150	6 ± 0.14	6 ± 0.3	12 ± 0	10 ± 0.03
Co(II)	50	0	0	0	0
	100	0	0	0	0
	150	5 ± 0.14	7 ± 0.06	7 ± 0.14	0
Ni(II)	50	0	0	11 ± 0	6 ± 0.14
	100	0	6 ± 0.14	13 ± 0	8 ± 0.14
	150	6 ± 0.06	8 ± 0.14	15 ± 0	10 ± 0.03
Mn(II)	50	0	0	0	0
	100	0	0	6 ± 0.03	0
	150	7 ± 0.14	0	8 ± 0.03	6 ± 0.14
UO_2_(II)	50	0	0	0	0
	100	0	0	7 ± 0.03	0
	150	6 ± 0.14	6 ± 0.14	9 ± 0.03	6 ± 0.06
Penicillin G	50	8 ± 0	10 ± 0.03	9 ± 0.06	0
	100	10 ± 0	12 ± 0.03	10 ± 0.03	0
	150	13 ± 0	16 ± 0	12 ± 0.03	0

**Table 3:** Antifungal activity of Schiff base and their metal mixed Schiff base complexes

Antifungal agent	Concentration(µg/ml)	Fungi		
		*Aspergillus niger*	*Fusarium oxysporum*	*Candida albicans*
Ligand	50	9 ± 0.14	0	8 ± 0.03
	100	11 ± 0.14	6 ± 0.14	10 ± 0.03
	150	13 ± 0.14	9 ± 0.14	12 ± 0
Cu(II)	50	9 ± 0.06	6 ± 0.14	6 ± 0.03
	100	11 ± 0.06	8 ± 0.14	9 ± 0
	150	13 ± 0.06	10 ± 0.14	11 ± 0
Co(II)	50	11 ± 0.14	7 ± 0.14	9 ± 0.03
	100	13 ± 0.03	10 ± 0.06	11 ± 0.03
	150	15 ± 0.03	12 ± 0.06	13 ± 0
Ni(II)	50	12 ± 0.03	6 ± 0.14	11 ± 0
	100	15 ± 0.03	8 ± 0.14	13 ± 0
	150	17 ± 0.03	10 ± 0.06	15 ± 0
Mn(II)	50	7 ± 0.14	0	9 ± 0.06
	100	10 ± 0.14	6 ± 0.14	11 ± 0.03
	150	12 ± 0.14	9 ± 0.14	13 ± 0.03
UO_2_(II)	50	10 ± 0.14	8 ± 0.06	7 ± 0.14
	100	12 ± 0.14	10 ± 0.06	11 ± 0.14
	150	14 ± 0.03	12 ± 0.03	14 ± 0.06
Miconazole	50	10 ± 0.03	10 ± 0.06	8 ± 0.03
	100	13 ± 0.03	12 ± 0.06	11 ± 0
	150	17 ± 0.03	14 ± 0.03	15 ± 0

Additionally, the Ni(II) complex exhibited greater antifungal activity than the other evaluated complexes and was comparable to the common antifungal, miconazole. The zone of inhibition (ZOI) for *F. oxysporum* was lower than that of the other examined fungi. A low concentration (50 µg/ml) of the Schiff base and Mn(II) complex showed no inhibition against *F. oxysporum*. High concentrations of the metal complexes (150 µg/ml) had detrimental effects on *A. niger* and *C. albicans*, with ZOI comparable to those of the common antifungal, miconazole. DMF demonstrated no inhibitory zones against any of the tested fungi. The findings align with earlier research, confirming the potent antibacterial properties of the produced compounds. The activity data obtained by Vijayalakshmi [52] indicated that the metal complexes were more effective against one or more bacterial species than the parent Schiff base ligand. Diab et al. [53]. The inhibition zones varied from 9 ± 0 to 35 ± 0 mm. However, Mohammed et al. [21] examined the biological activity of 4-amino antipyrines and their complexes against various organisms, including fungi such as *A. niger* and *A. flavus* and bacteria such as Gram-positive *S. aureus* and Gram-negative *E. coli* and *K. pneumoniae*. They found high inhibition zones ranging from 20 to 70 mm.

Analyzing the data closely shows that, depending on the type of metal, the metal complex analogues of the produced Schiff base ligand exhibit different ranges of minimal inhibition. Schiff bases, recognized as versatile pharmacophores for the design and development of various physiologically active drugs, were initially described by Hugo Schiff when he reacted amines and carbonyl compounds with an azomethine (>C=N–) functional group [54, 55]. It has been found that the carbon–nitrogen double bond in these compounds is essential to their biological properties [56]. The nitrogen of the azomethine group (>C=N) serves as an effective donor site due to the presence of a lone pair of electrons on the nitrogen atom, the electron-donating nature of the double bond, and the low electronegativity of nitrogen. This characteristic makes Schiff bases biologically active agents against fungal infections, free radicals, malaria, viruses, cancer, bacterial infections, inflammation, and fever [57, 58]. The bonding methods and chelating abilities of these compounds toward the central metal atom contribute to their unique properties. The imine group (–N=CH–) can potentially serve as a donor for coordination. These versatile ligands have a variety of chelating properties and biological functions. In addition, recent years have highlighted the role of free oxygen radicals in experimental studies. The physiological activity of cellular responses to anoxia, which acts as a defense against infectious diseases, illustrates the beneficial effects of reactive oxygen species (ROS). Despite this advantage, new research suggests that radicals generated during biological-organic oxidation–reduction processes may lead to oxidative damage in various body parts and contribute to the development of mutations [59]. As Schiff base ligands possess different donor atoms and diverse modes of attachment to metal ions, they exhibit a wide range of biological activities. By modifying the substituents, which can vary the final donating atoms, these interactions create an intriguing series of ligands with characteristics that can be tailored to produce distinct compounds.

Bioactivity against bacteria at low concentrations was preferentially induced by the presence of distinct functional groups on the benzene ring, showing increased efficacy against *F. oxysporum*, *A. niger*, *C. albicans*, *B. cereus*, *S. aureus*, *P. aeruginosa*, and *E. coli*. The inhibition of normal cell processes caused by hydrogen bond formation through active centers in cell components with >CH=N– may be linked to the disruption of cell wall production [60, 61].

### MIC and MMC studies

**Fig. 2:**
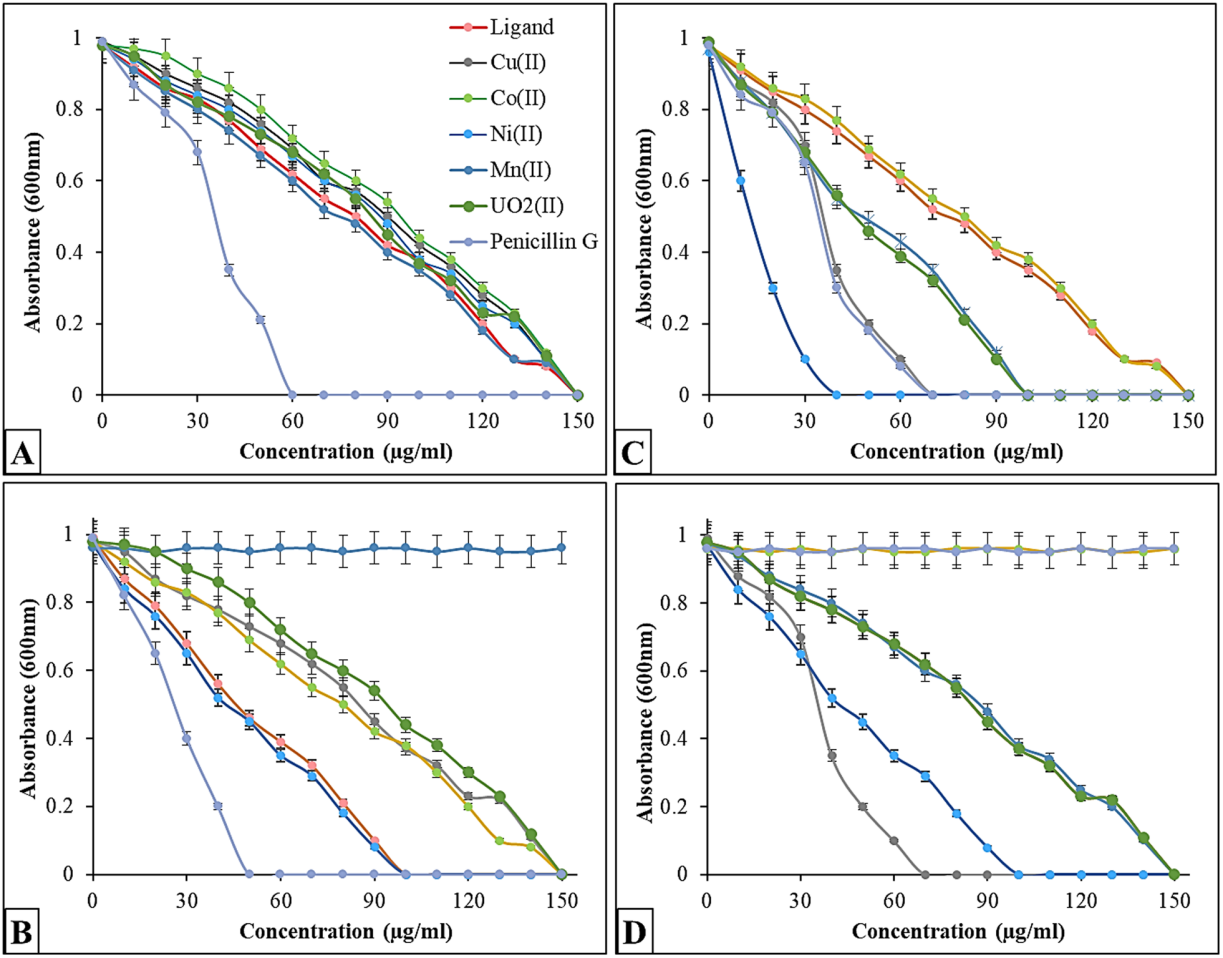
Minimum inhibition concentration of Schiff base ligand and its metal complexes compared to penicillin G against *B. cereus*; (**A**), *S. aureus*; (**B**), *E. coli*; (**C**), and *P. aeruginosa*; (**D**)

**Fig. 3:**
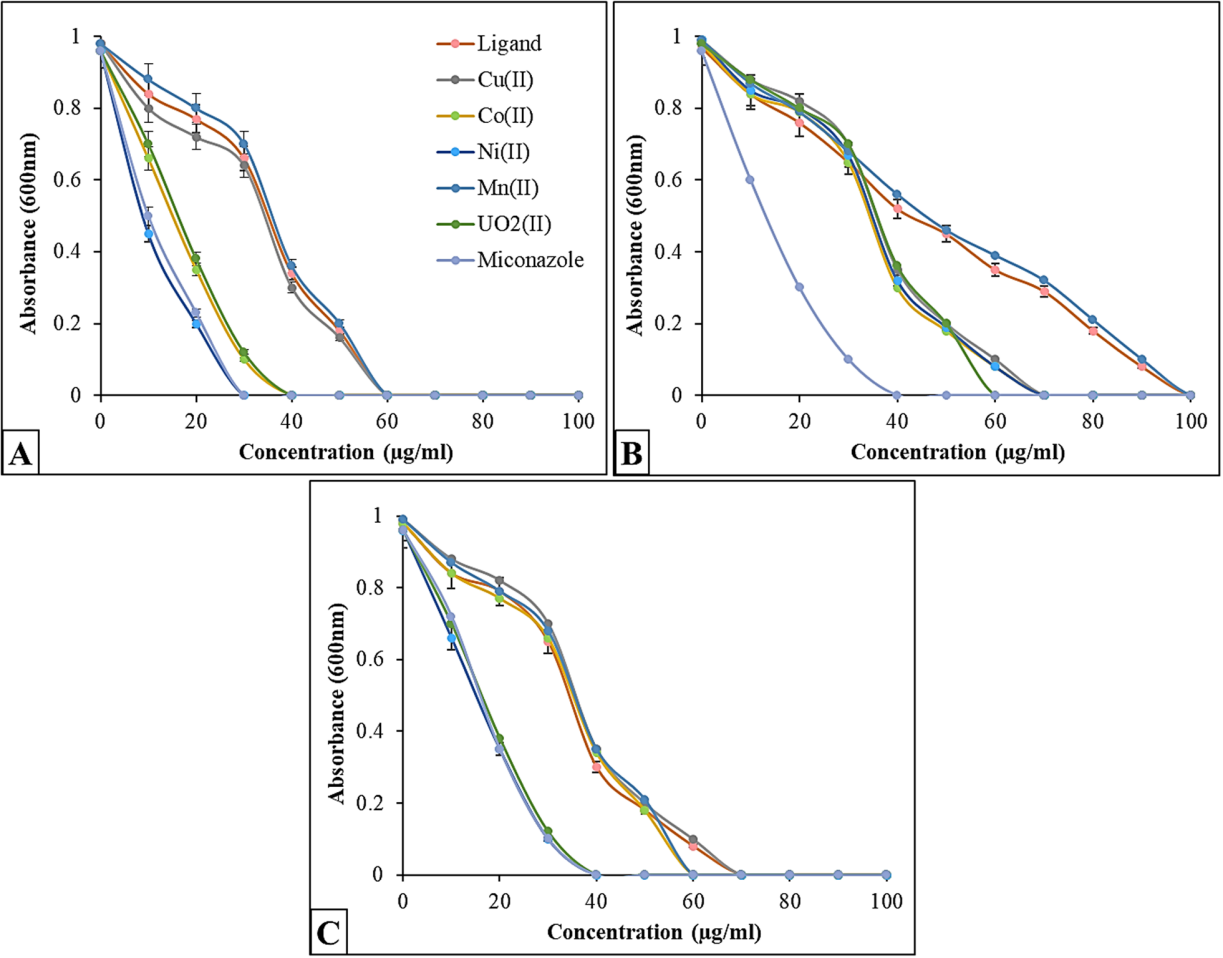
Minimum inhibition concentration of Schiff base ligand and its metal complexes compared to miconazole against *Aspergillus niger*; (**A**), *Fusarium oxysporum*; (**B**), and *Candida albicans*; (**C**)

The Schiff base ligand and its metal complexes were tested against bacteria that are Gram-positive (*B. cereus* and *S. aureus*), Gram-negative bacteria (*E. coli* and *P. aeruginosa*), and fungi (*A. niger*, *F. oxysporum*, and *C. albicans*) at doses ranging from 10 to 150 µg/ml (Figs. 2 and 3). The concentration that results in full inhibition (no discernible microbial growth) is known as the MIC value. As the concentration of each studied molecule increased, so did its biocidal effect [62]. In contrast to the Schiff base ligand and other complexes, a dosage of 30–100 µg/ml of complex (3) was sufficient to completely inhibit most microbial strains, except *B. cereus* (150 µg/ml), demonstrating its potent antibacterial and antifungal activities. With regard to *E. coli*, the Ni(II) complex displayed the strongest antibacterial activity, followed by Cu(II), UO2(II), Mn(II), and Co(II) complexes. The MIC values for the Schiff base ligand and Ni(II) complex against *B. cereus* and *S. aureus* were identical. *A. niger* and *C. albicans* were inhibited by 70 µg/ml of all prepared complexes. Against the studied *B. cereus*, only high doses of metal complexes (≥140 µg/ml) showed mild to moderate antibacterial activity. MMC was defined as the lowest concentration yielding no growth of microorganisms on subculture. Barakat et al. [63] indicated that the MIC and MMC of 4-antipyrine and its metal complexes were ≈60 and ≈512 µg/ml, respectively, indicating excellent to moderate activity. The MIC values of 4-amino antipyrine and its complexes ranged from 20 to 55 μg/ml [21].

### Antioxidant assay

**Fig. 4:**
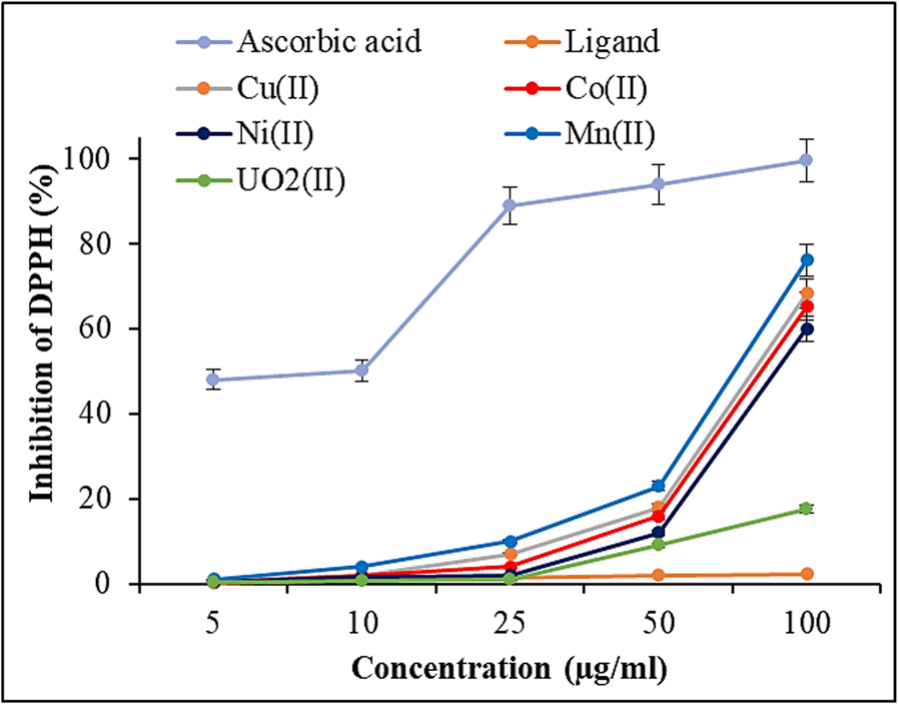
DPPH scavenging activity of different concentrations Schiff base ligand and its metal complexes compared to the standard ascorbic acid

**Fig. 5:**
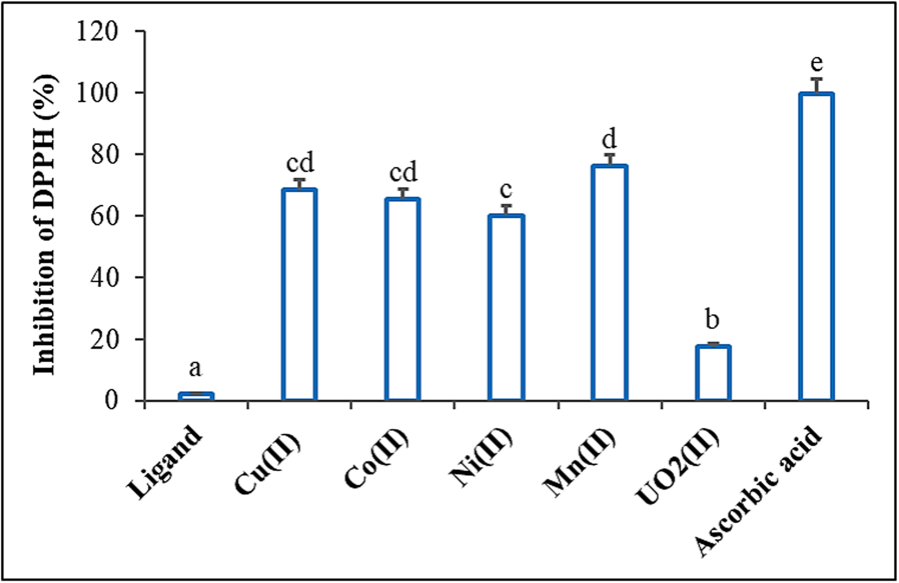
DPPH scavenging activity of Schiff base ligand and its metal complexes. Vertical bars represent the SD. Means denoted by similar letter are not significantly different at *p* ≤ 0.05 using LSD test

Using the in vitro DPPH radical scavenging technique, we assessed the antioxidant properties of the Schiff base and its metal complexes (Figs. 4, 5). The findings demonstrated that the chemical and its metal complexes exhibited a concentration-dependent scavenging effect, with the radical scavenging ratio increasing as the concentrations within the tested range rose. The DPPH radical scavenging activities were 2.28% for the Schiff base, 68.4% for the Cu(II) complex, 65.3% for the Co(II) complex, 60.1% for the Ni(II) complex, 76.2% for the Mn(II) complex, and 17.6% for the UO_2_(II) complex. At a concentration of 100 µg/ml, the highest free radical scavenging activity was observed in the Mn(II) complex (76.2%), while the lowest was in the UO_2_(II) complex (17.6%). In this study, ascorbic acid, serving as a standard antioxidant, demonstrated greater activity than the other compounds at all concentrations. Furthermore, the experimental results support the previously reported study [64–66].

The reversible interaction between DPPH and compounds with hydroxyl groups, such as phenols, may account for the reported variations in the scavenging capabilities of the produced compounds against the DPPH radical, which explains the low antioxidant activity values. The moderate inhibition of the DPPH radical suggests that the analogues of the metal complexes of the prepared Schiff base ligand possess a strong ability to scavenge free radicals, despite the results of the DPPH free radical-scavenging assay for the synthesized compounds being lower than those for the reference drug ascorbic acid.

The structure–activity relationship of the produced compounds is partially revealed through antibacterial, antifungal, and antioxidant assessments. The higher activity and reported biological characteristics of the active compounds may result from the hydroxyl and azomethine groups (>C=N), depending on the substituent type. These metal complexes have the potential to serve as a powerful source for developing chemotherapeutic and therapeutic medicines for treating pathological diseases caused by stress.

### Molecular docking study

In molecular docking, the S value (also referred to as the docking score) represents the estimated binding free energy between a ligand and its target protein. Lower (more negative) S values indicate stronger binding affinities, as they reflect more favorable interactions and greater binding stability. These negative values arise because binding typically releases energy (an exergonic process), meaning the ligand–protein complex is thermodynamically more stable than the unbound forms. Against *C. albicans*, Ni achieved the lowest Score value (S = −9.28784 kcal/mol), indicating superior binding (Table 4).For *A. niger*, Ni (S = −6.88357 kcal/mol) and U (S = −6.43079 kcal/mol) showed strong affinity (Table 5).Against *F.oxysporum*, U (S = −6.06245 kcal/mol) and Co (S = −5.8091 kcal/mol) were most effective (Table 6).

**Table 4:** Docking scores and energies of ligand N and its complexes with crystal structure of *Candida albicans* (PDB ID 5V5Z)

Mol	Docking score kcal/mol (S)	rmsd_refine	E_conf	E_place	E_score1	E_refine	E_score2
Ligand	−6.04214	0.8016086	1.203483	−73.8604	−10.5174	−23.4871	−6.04214
Cu(II)	−5.15878	2.2565432	−297.408	−27.6371	−4.13051	−33.4374	−5.15878
Co(II)	−7.02663	2.5974302	−530.712	−28.6023	−7.65268	−2.85538	−7.02663
Ni(II)	−9.28784	1.5827925	−710.212	−45.9122	−8.09957	−14.3363	−9.28784
Mn(II)	−6.86913	2.4570975	−663.462	44.93802	−3.69879	3.017609	−6.86913
UO_2_(II)	−5.8841	2.4298897	−1607.08	−77.6064	−12.8032	43.26406	−5.8841

**Table 5:** Docking scores and energies of ligand N and its complexes with crystal structure of *Aspergillus niger* (PDB ID 3PL3)

Mol	Docking Score kcal/mol (S)	rmsd_refine	E_conf	E_place	E_score1	E_refine	E_score2
Ligand	−6.3869	1.0032259	−2.22955	−81.7163	−12.793	−36.8238	−6.3869
Cu(II)	−6.24181	3.2930138	−326.673	0.526862	−7.25623	−34.1277	−6.24181
Co(II)	−6.51437	2.1607237	−697.063	−6.49945	−6.39737	−26.5237	−6.51437
Ni(II)	−6.88357	2.561784	−731.91	−50.8112	−9.37493	−2.38053	−6.88357
Mn(II)	−5.92222	3.5039871	−779.722	−53.6052	−6.12147	−32.8969	−5.92222
UO_2_(II)	−6.43079	2.2323217	−1920.09	−57.52	−10.6568	−15.7126	−6.43079

**Table 6:** Docking scores and energies of ligand N and its complexes with crystal structure of *Fusarium oxysporum* (PDB ID 1FN8)

Mol	Docking Score kcal/mol (S)	rmsd_refine	E_conf	E_place	E_score1	E_refine	E_score2
Ligand	−1.80357	2.16103	−1.46626	−58.6385	−10.2807	−21.1839	−1.80357
Cu(II)	−5.63574	3.313546	−320.125	−33.466	−9.05757	−32.5768	−5.63574
Co(II)	−5.8091	2.792679	−708.828	−34.1028	−10.1414	−29.6237	−5.8091
Ni(II)	−5.11943	3.387218	−768.002	−24.5664	−7.85059	−20.3122	−5.11943
Mn(II)	−1.94296	2.996786	−780.105	−3.17873	−6.41153	−30.7763	−1.94296
UO_2_(II)	−6.06245	2.719456	−2035.69	−33.0997	−9.13752	−24.3461	−6.06245

Interaction Analysis:

- Ni formed a strong H-donor interaction with OE1 GLU 209 (−3.7 kcal/mol) in *A. niger* (Table 7).
- Mn exhibited the strongest H-donor bond (−4.4 kcal/mol) with O PRO 462 in *C. albicans* (Table 8).
- π–H and π-cation interactions contributed significantly, e.g., 6-ring of Ni with NH_2_ ARG 248 (−0.5 kcal/mol, Table 9).

**Table 7:** Interaction of ligand N and its complexes with crystal structure of *Candida albicans* (PDB ID 5V5Z)

Mol	Atom involved	Residue	Interaction type	Distance (Å)	Binding energy (kcal/mol)
Ligand	O 26	O GLY 303 (A)	H-donor	2.72	−1.8
Cu(II)	O 28	SG CYS 470 (A)	H-acceptor	3.15	−0.9
	6-ring	CD1 LEU 376 (A)	pi-H	3.84	−0.6
	6-ring	CA PHE 463 (A)	pi-H	4.17	−0.7
Co(II)	O 52	O HIS 468 (A)	H-donor	2.92	−1.4
Ni(II)	O 54	CE2 TYR 132 (A)	H-acceptor	3.15	−2.2
Mn(II)	O 14	O PRO 462 (A)	H-donor	2.66	−4.4
UO_2_(II)	N 40	SG CYS 470 (A)	H-acceptor	3.30	−0.8
	O 54	OH TYR 132 (A)	H-acceptor	3.47	−0.5
	O 54	CB CYS 470 (A)	H-acceptor	3.11	−0.9
	6-ring	CA PHE 463 (A)	pi-H	4.20	−1.0

H-donor (Hydrogen donor): Atom or group that donates a hydrogen atom to form a hydrogen bond with an acceptor (e.g., O or N)

H-acceptor: Atom that accepts a hydrogen bond from a donor

π–H (pi-hydrogen interaction): A weak non-covalent interaction between a hydrogen atom and the π-electron cloud of an aromatic ring

π-cation: A stabilizing electrostatic interaction between a positively charged ion (cation) and an aromatic π-system

Ionic interaction: An electrostatic attraction between oppositely charged ions or groups (e.g., a metal ion and a negatively charged amino acid side chain

**Table 8:** Interaction of ligand N and its complexes with crystal structure of *Aspergillus niger* (PDB ID 3PL3)

Mol	Atom involved	Residue	Interaction type	Distance (Å)	Binding energy (kcal/mol)
Ligand	O 22	OD2 ASP 343 (A)	H-donor	3.08	−1.1
	O 26	OE1 GLU 214 (A)	H-donor	2.89	−3.0
	6-ring	CA ASP 256 (A)	pi-H	4.66	−0.5
Cu(II)	6-ring	CE2 TYR 249 (A)	pi-H	3.65	−0.5
	6-ring	CB PRO 386 (A)	pi-H	3.73	−0.5
Co(II)	O 41	OD1 ASP 247 (A)	H-donor	3.54	−0.8
Ni(II)	O 52	OE1 GLU 209 (A)	H-donor	2.97	−3.7
	O 52	OE2 GLU 209 (A)	H-donor	2.89	−2.2
	O 54	NH1 ARG 104 (A)	Ionic	3.87	−0.8
	6-ring	NH2 ARG 248 (A)	pi-cation	4.83	−0.5
	6-ring	5-ring TRP 371 (A)	pi-pi	3.45	−0.1
Mn(II)	O 41	O THR 243 (A)	H-donor	3.19	−0.8
	6-ring	CA ASN 246 (A)	pi-H	3.81	−1.7
UO_2_(II)	O 54	CG PRO 386 (A)	H-acceptor	3.43	−0.8

H-donor (Hydrogen donor): Atom or group that donates a hydrogen atom to form a hydrogen bond with an acceptor (e.g., O or N)

H-acceptor: Atom that accepts a hydrogen bond from a donor

π–H (pi-hydrogen interaction): A weak non-covalent interaction between a hydrogen atom and the π-electron cloud of an aromatic ring

π-cation: A stabilizing electrostatic interaction between a positively charged ion (cation) and an aromatic π-system

Ionic interaction: An electrostatic attraction between oppositely charged ions or groups (e.g., a metal ion and a negatively charged amino acid side chain

**Table 9:** Interaction of ligand N and its complexes with crystal structure of *Fusarium oxysporum* (PDB ID 1FN8)

Mol	Atom involved	Residue	Interaction type	Distance (Å)	Binding energy (kcal/mol)
Ligand	O 24	NH2 ARG 73 (A)	H-acceptor	3.11	−0.8
	6-ring	CA SER 150 (A)	pi-H	3.81	−0.9
Cu(II)	O 52	O GLY 148 (A)	H-donor	2.84	−3.7
Co(II)	O 14	O TRP 41 (A)	H-donor	2.85	−3.4
	N 51	N THR 151 (A)	H-acceptor	3.25	−3.1
	6-ring	NH2 ARG 73 (A)	pi-cation	3.62	−0.8
Ni(II)	O 41	O GLY 148 (A)	H-donor	2.93	−2.9
	6-ring	N THR 151 (A)	pi-H	3.85	−0.6
Mn(II)	O 41	O TRP 41 (A)	H-donor	2.84	−2.2
	O 54	O PRO 40 (A)	H-donor	2.78	−3.7
UO_2_(II)	O 29	OE1 GLN 192 (A)	H-donor	2.89	−2.5

H-donor (Hydrogen donor): Atom or group that donates a hydrogen atom to form a hydrogen bond with an acceptor (e.g., O or N)

H-acceptor: Atom that accepts a hydrogen bond from a donor

π–H (pi-hydrogen interaction): A weak non-covalent interaction between a hydrogen atom and the π-electron cloud of an aromatic ring

π-cation: A stabilizing electrostatic interaction between a positively charged ion (cation) and an aromatic π-system

Ionic interaction: An electrostatic attraction between oppositely charged ions or groups (e.g., a metal ion and a negatively charged amino acid side chain

**Fig. 6:**
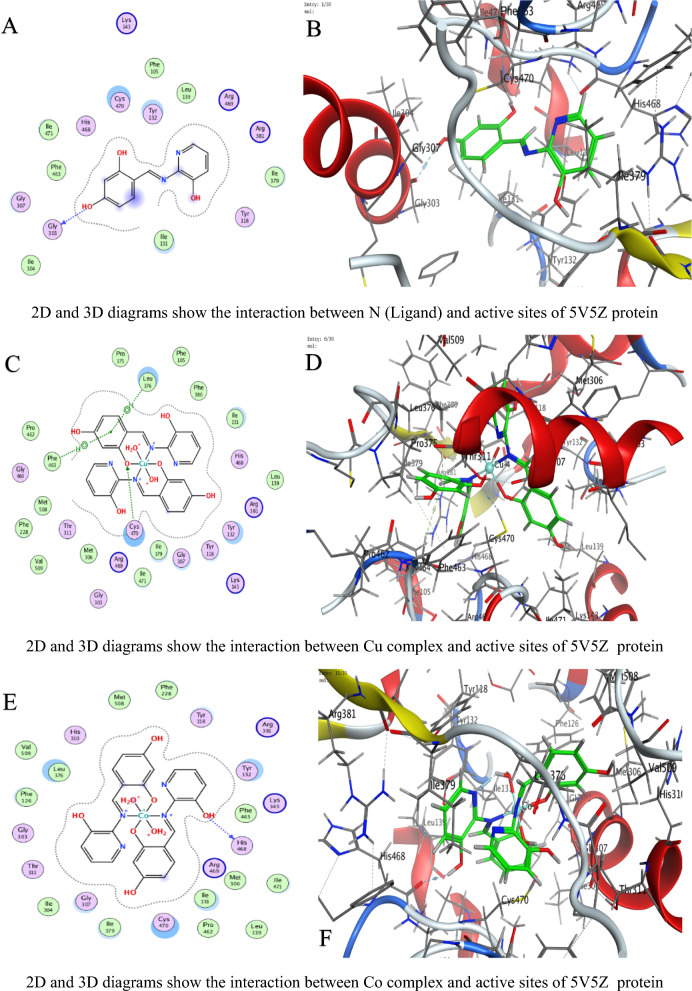

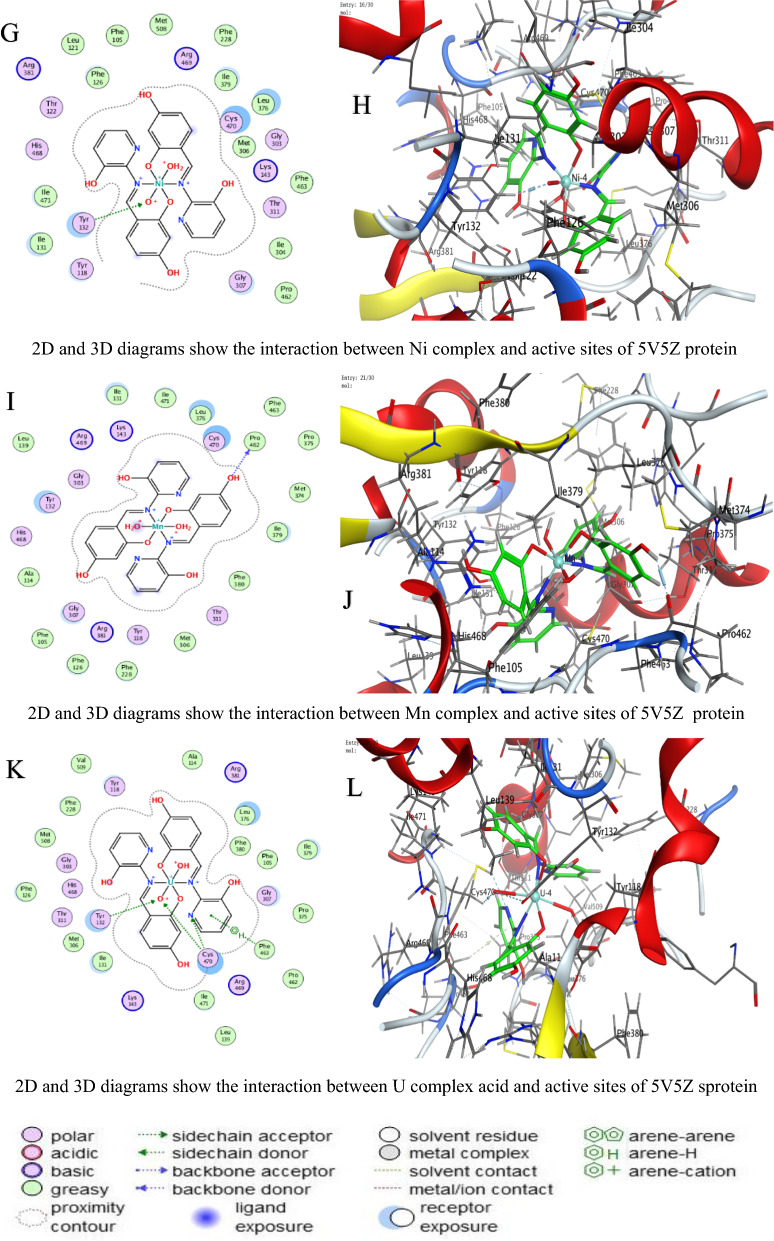
The representative key for the types of interaction between ligands and *Candida albicans* PDB ID: 5V5Z protein

**Fig. 7:**
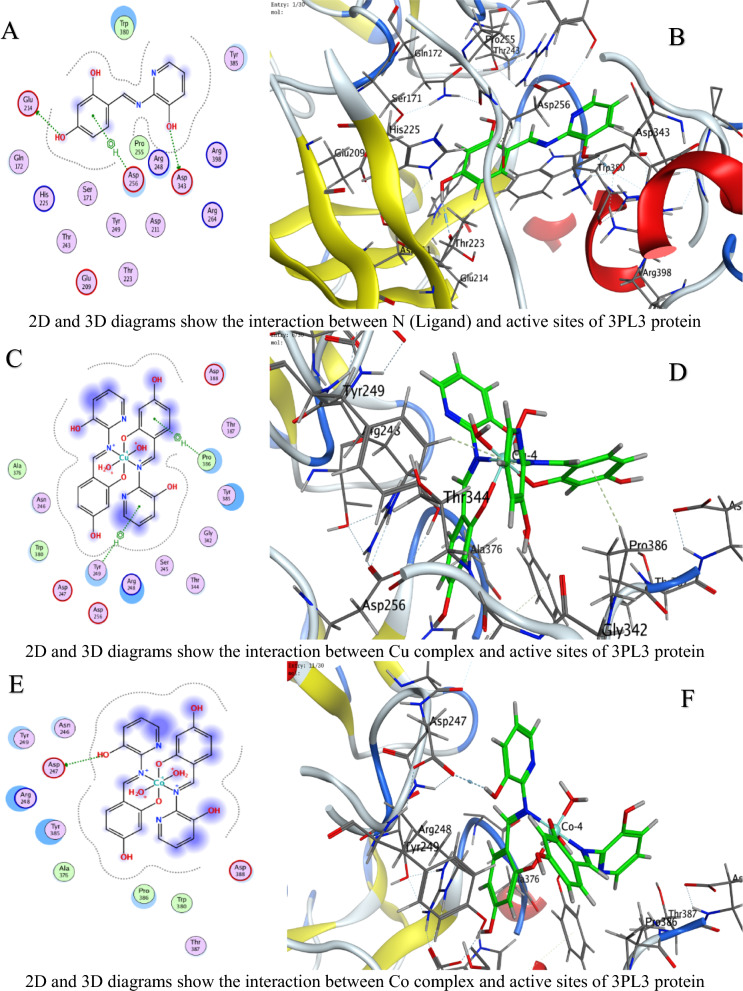

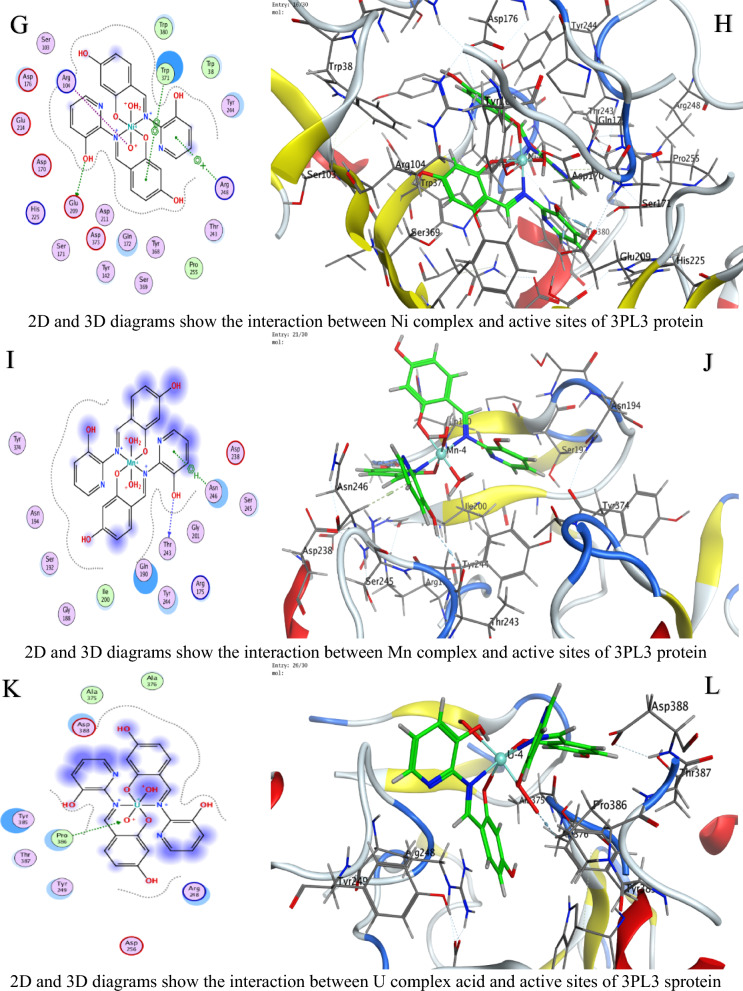

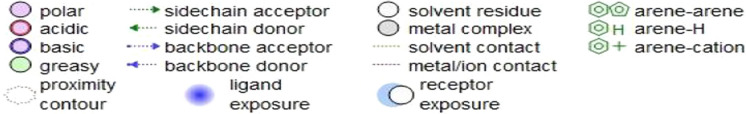
The representative key for the types of interaction between ligands and *Aspergillus niger* PDB ID: 3PL3 protein

**Fig. 8:**
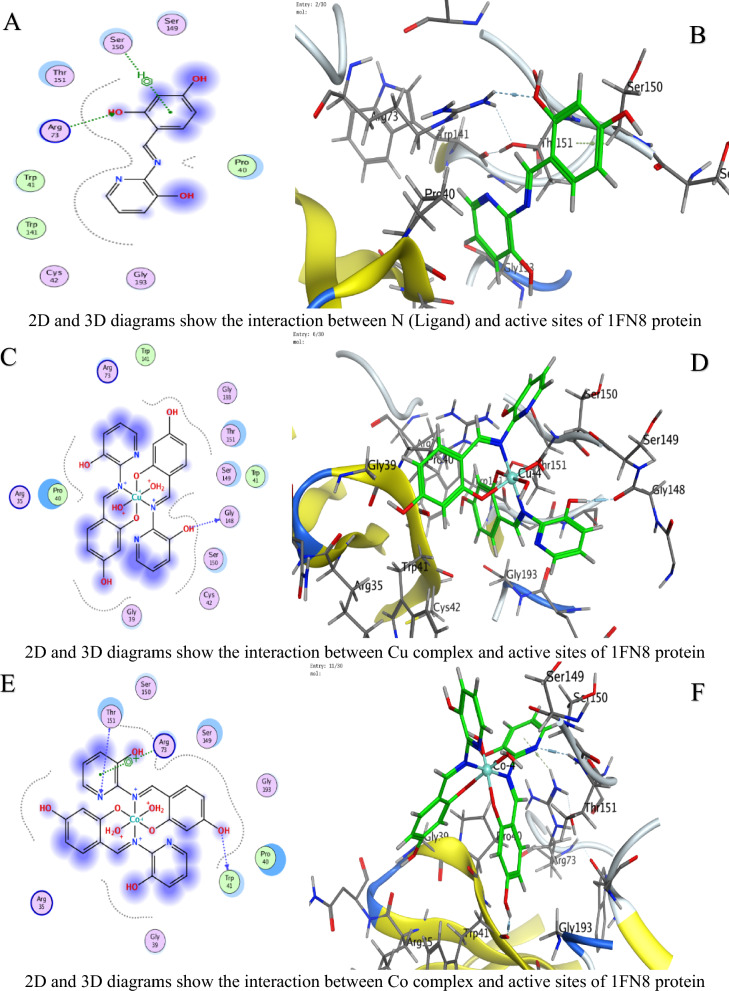

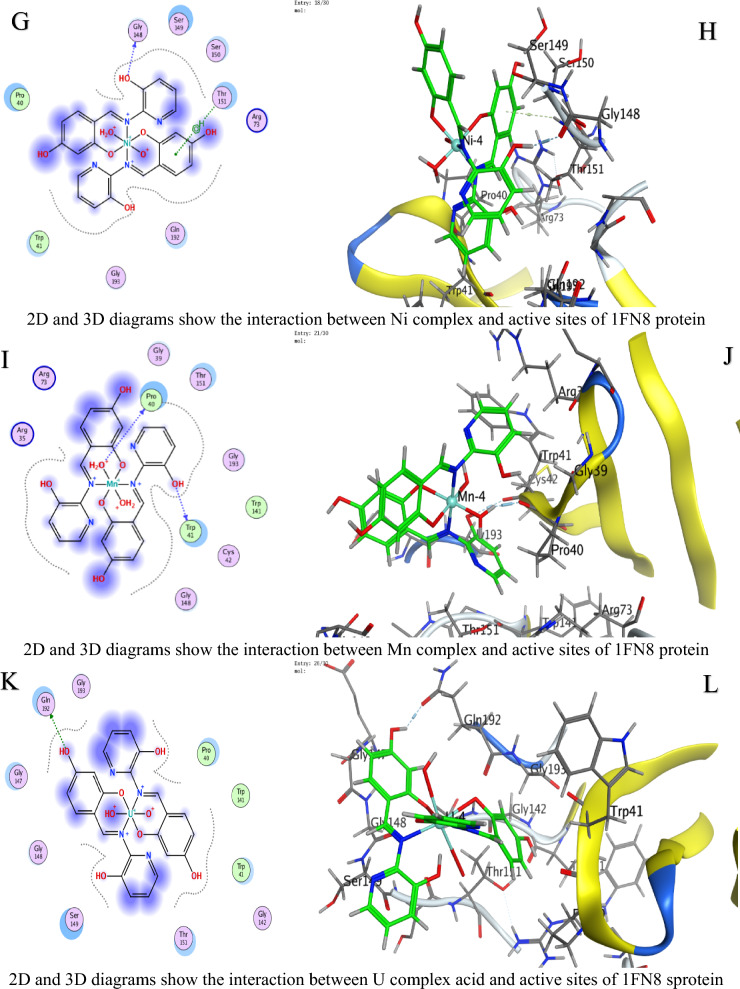

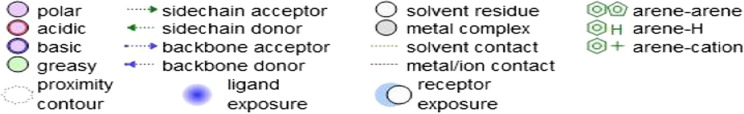
The representative key for the types of interaction between ligands and *Fusarium oxysporum* PDB ID:1FN8 protein

Table 10 is a summary table that shows all S-scores and E_refine values for easy comparison. The best-fitted 2D and 3D poses chosen by the ligand and its metal complexes are represented in Figs. 6, 7, and 8.The superior performance of Ni complex aligns with prior studies demonstrating nickel’s ability to disrupt fungal metalloenzymes [67]. The greater potency of the Ni complex against *C. albicans* (S = −9.28784 kcal/mol, Table 4) likely stems from its dual ability to form strong hydrogen bonds (e.g., −3.7 kcal/mol with OE1 GLU 209 in *A. niger*, Table 8) and stabilize π-cation interactions (e.g., with NH2 ARG 248). These interactions align with prior findings that nickel disrupts fungal metalloenzymes by competing with native metal ions in catalytic sites [68]. However, complex (3) reduced efficacy in *A. niger* (S = −6.88357kcal/mol, Table 5) may reflect differences in the active site architecture, such as the absence of complementary residues for π-cation bonding or steric hindrance from bulkier side chains (e.g., PRO 386 in Table 8).

**Table 10:** A summary table of all S-scores and E_refine values

Mol	Protein	Docking score kcal/mol (S)	E_refine
Ligand	5V5Z	−6.04214	−23.4871
Cu(II)	5V5Z	−5.15878	−33.4374
Co(II)	5V5Z	−7.02663	−2.85538
Ni(II)	5V5Z	−9.28784	−14.3363
Mn(II)	5V5Z	−6.86913	3.017609
UO_2_(II)	5V5Z	−5.8841	43.26406
Ligand	3PL3	−6.3869	−36.8238
Cu(II)	3PL3	−6.24181	−34.1277
Co(II)	3PL3	−6.51437	−26.5237
Ni(II)	3PL3	−6.88357	−2.38053
Mn(II)	3PL3	−5.92222	−32.8969
UO_2_(II)	3PL3	−6.43079	−15.7126
Ligand	1FN8	−1.80357	−21.1839
Cu(II)	1FN8	−5.63574	−32.5768
Co(II)	1FN8	−5.8091	−29.6237
Ni(II)	1FN8	−5.11943	−20.3122
Mn(II)	1FN8	−1.94296	−30.7763
UO_2_(II)	1FN8	−6.06245	−24.3461

UO_2_ Complex notable affinity for *F. oxysporum* (S = −6.06245 kcal/mol, Table 5) could arise from its unique ionic radius and electrophilicity, enabling unconventional interactions with sulfur-containing residues like SG CYS 470 (Table 9). This aligns with studies suggesting heavy metals disrupt redox balance in fungi by binding to cysteine-rich regions [68]. Despite its moderate S score, complex (5) multi-modal interactions (e.g., H-acceptor with CG PRO 386 and π–H with CA PHE 463) highlight its potential for broad-spectrum targeting.

Variations in RMSD values reflect differences in docking precision. Ni complex displayed the lowest RMSD (1.58 Å) in *C. albicans*, indicating a well-optimized pose. In contrast, Cu’s higher RMSD (3.29 Å) in *A. niger* suggests less stable binding, likely due to weaker π–H interactions and suboptimal placement (E_place). The strong H-donor interactions observed for Mn and Ni complexes correlate with their low E_refine values, suggesting stable binding conformations [15, 69–73]. Notably, uranium’s affinity for *F.oxysporum* could relate to its unique electrostatic interactions with SG CYS 470, a residue critical for fungal redox balance (Table 9) [68, 74]. The positive E_place value for Mn complex in *C. albicans* (44.93802, Table 4) implies unfavorable initial ligand placement, but its strong H-donor interaction (−4.4 kcal/mol, Table 7) compensates, emphasizing the role of post-placement refinement in the docking scoring procedure [75, 76].

Energy term analysis highlights compromises. Energy analyses further support these trends. For instance, Mn's strong hydrogen bonding (−4.4 kcal/mol) in *C. albicans* partially compensates for its poor initial placement (E_place = 44.94). Similarly, low E_conf values (e.g., −697.06 for *A. niger*) confirm conformational stability, though suboptimal solvation can affect the overall S score (Table 10).

## Conclusions

Condensation of 2,4-dihydroxybenzaldehyde and 2-amino-3-hydroxypyridine yielded a Schiff base ligand. The elemental analyses and FT-IR spectra data of the ligand and its complexes were used for the characterization of the structures. The Schiff base ligand and its bivalent transition metal complexes were screened for their antimicrobial activity against Gram-positive, Gram-negative bacterial and fungal strains; complex (3) showed enhanced activities against both Gram-positive and Gram-negative bacterial strains as well as fungal strains compared with other complexes and standard drug penicillin G. Complex (4) demonstrated the greatest inhibition of DPPH radicals, with a %DPPH ihibition value of 76.2%, according to antiradical screening of the compounds against DPPH free radicals. The computational results obtaineddemonstrate significant alignment with in vitro findings conducted in this study. The ligand and its metal complexes, particularly Ni(II) and UO_2_(II), demonstrate promising antifungal potential via strong docking scores and diverse interactions. These results support further exploration of metal-based antifungals, with emphasis on optimizing π–H and H-bond networks. According to the findings of the biological investigations, these metal complexes may be used as building blocks for the creation of new chemotherapeutic drugs to treat pathological diseases brought on by stress that are linked to the production of radicals and the regulation of disease. In vitro model studies, cytotoxicity, and stability elucidations should be investigated in future work.Therefore, a much-needed screening strategy and high-throughput toxicity testing methodologies that are necessary for the initial risk evaluation of Schiff base ligands and their bivalent transition metal complexes are now based on pertinent in vitro toxicological models based on recognized cell lines. To ensure the accuracy of the data obtained and potentially establish a structure–toxicity relationship, it is important to note that all toxicity data must be interpreted in light of the physicochemical properties of the antimicrobial materials, by*in vitro* toxicology. Then,stable antimicrobial materialsmay provide further therapeutic benefits in different applications.

## Data Availability

All relevant data are within the manuscript and available from the corresponding author upon request.

## Abbreviations

DPPH: 2,2-Diphenyl-1-picrylhydrazyl
MOE: Molecular operating environment
MIC: Minimum inhibition concentration
MMC: Minimum microbicidal concentration
DMF: Dimethylformamide
ZOI: Zone of inhibition
NBM: Nutrient broth medium
NAM: Nutrient agar medium
CFU: Colony-forming unit
FT-IR: Fourier-transform infrared spectroscopy
E_place: Score of the placement phase
E_conf: Energy conformer
E_refine: Score refinement
SD: Standard deviation
